# Chicago Veterinary College

**Published:** 1890-09

**Authors:** 


*Chicago Veterinary College.*—In this age of progress, especially in veterinary matters, there is nothing more important nor of greater interest than veterinary education and the facilities of acquiring it. It is difficult to make progress in scientific study without suitable accommodations, appliances and material. The schools should be large enough to accommodate the students without crowding, both in the interests of progress and the health of the class.

In the Chicago Veterinary College we find a good illustration of the progress of veterinary science keeping pace with the development of the country and its live stock interests. It was started in 1883, in small rented quarters, which it outgrew in three years. In 1886 a two-story building, 50x146 feet was erected by the trustees, which was thought at that time to be ample to accommodate all the classes for a long time, but the building was found to be too small for the class of 1888-9. The class of 1889-90 found the building they had left on the last day of March had grown immensely during the Summer; another story had been added, more than doubling its former capacity. The former hospital had been given over entirely to the driving horses of the resident practitioners. The hospital, much enlarged, had gone on to the second floor, the old lecture hall had been transformed into a chemical laboratory, and the new story contained a handsome amphitheatral lecture hall, with a seating capacity of three hundred, a dissecting room, 50x80 feet, with the ceiling 16 feet above the floor, and a museum. The microscopical laboratory and operating room were untouched. The growth and changes in a few months had rendered the building almost unrecognizable. At present it affords ample facilities for the demands of the times, but future prospects indicate that further additions will have to be made to keep pace with the rapid development of the Western part of the country.

